# Supplementary material for: Robust disease prognosis via diagnostic knowledge preservation: A sequential learning approach
Source: PLoS One. 2026 May 6;21(5):e0344600. doi: 10.1371/journal.pone.0344600 (PMC13148697; doi:10.1371/journal.pone.0344600)
Supplement: S1 Table — (DOCX) [file pone.0344600.s002.docx]

**S1 Table.** Patient Characteristics in Structural Progression OAI and MOST Cohorts.

| **Dataset** | **Parameters** | **Men** | | **Women** | |
| --- | --- | --- | --- | --- | --- |
|  |  | **Patients** | **Controls** | **Patients** | **Controls** |
| **OAI Dataset** | No. of patients | 216 | 707 | 355 | 970 |
|  | No. of scans | 241 | 917 | 421 | 1348 |
|  | Mean age (y) | 62.3±9.0 | 62.1±9.3 | 63.7±8.2 | 62.5±8.9 |
|  | Mean height (m) | 1.8±0.1 | 1.8±0.1 | 1.6±0.1 | 1.6±0.1 |
|  | Mean weight (kg) | 93.1±14.3 | 91.7±14.4 | 80.8±14.8 | 78.2±14.5 |
|  | Mean BMI (kg/m²) | 30.1±4.0 | 29.3±4.0 | 30.6±5.2 | 29.6±5.3 |
|  | **Ethnicity** | | | | |
|  | White | 184 | 595 | 253 | 721 |
|  | Black | 26 | 97 | 90 | 230 |
|  | Asian | 2 | 4 | 3 | 5 |
|  | Other nonwhite | 4 | 11 | 9 | 14 |
| **MOST Dataset** | No. of patients | 203 | 231 | 359 | 386 |
|  | No. of scans | 231 | 282 | 427 | 495 |
|  | Mean age (y) | 62.8±8.5 | 63.2±8.4 | 64.2±7.6 | 63.5±7.3 |
|  | Mean height (m) | 1.8±0.1 | 1.8±0.1 | 1.6±0.1 | 1.6±0.1 |
|  | Mean weight (kg) | 100.9±19.8 | 98.3±17.8 | 86.3±18.5 | 85.0±18.2 |
|  | Mean BMI (kg/m²) | 32.0±5.8 | 31.1±5.4 | 32.7±6.8 | 31.9±6.6 |
|  | **Ethnicity** | | | | |
|  | White | 177 | 197 | 288 | 302 |
|  | Black | 22 | 32 | 63 | 82 |
|  | Other | 4 | 2 | 8 | 2 |

**Note: Mean data are presented as mean±standard deviation. BMI = Body Mass Index, MOST= Multi-Center Osteoarthritis Study, OAI= Osteoarthritis Initiative. Number of patients are shown for ethnicity data.*
