# Supplementary material for: Robust disease prognosis via diagnostic knowledge preservation: A sequential learning approach
Source: PLoS One. 2026 May 6;21(5):e0344600. doi: 10.1371/journal.pone.0344600 (PMC13148697; doi:10.1371/journal.pone.0344600)
Supplement: S2 Table — (DOCX) [file pone.0344600.s003.docx]

**S2 Table**. Patient Characteristics in Structural Incidence OAI and MOST Cohorts

| **Dataset** | **Parameters** | **Men** | | **Women** | |
| --- | --- | --- | --- | --- | --- |
|  |  | **Patients** | **Controls** | **Patients** | **Controls** |
| **OAI Dataset** | No. of patients | 149 | 1103 | 271 | 1336 |
|  | No. of scans | 160 | 1737 | 296 | 2171 |
|  | Mean age (y) | 61.0±8.7 | 59.6±9.4 | 60.3±8.6 | 60.5±9.0 |
|  | Mean height (m) | 1.8±0.1 | 1.8±0.1 | 1.6±0.1 | 1.6±0.1 |
|  | Mean weight (kg) | 91.0±14.9 | 91.0±14.9 | 77.2±14.2 | 71.3±13.3 |
|  | Mean BMI (kg/m²) | 29.1±4.1 | 28.1±3.8 | 29.3±4.8 | 27.1±4.8 |
|  | **Ethnicity** | | | | |
|  | White | 133 | 975 | 208 | 1121 |
|  | Black | 13 | 110 | 56 | 180 |
|  | Asian | 1 | 4 | 4 | 13 |
|  | Other nonwhite | 2 | 12 | 2 | 21 |
| **MOST Dataset** | No. of patients | 179 | 600 | 326 | 795 |
|  | No. of scans | 196 | 945 | 371 | 1304 |
|  | Mean age (y) | 61.3±7.8 | 60.6±7.8 | 62.4±8.0 | 61.1±7.7 |
|  | Mean height (m) | 1.8±0.1 | 1.8±0.1 | 1.6±0.1 | 1.6±0.1 |
|  | Mean weight (kg) | 99.8±17.1 | 93.7±15.1 | 82.6±15.6 | 77.4±14.1 |
|  | Mean BMI (kg/m²) | 31.3±5.3 | 29.6±4.5 | 30.9±5.9 | 28.8±5.2 |
|  | **Ethnicity** | | | | |
|  | White | 157 | 573 | 270 | 694 |
|  | Black | 19 | 73 | 50 | 89 |
|  | Other | 3 | 10 | 3 | 12 |

**Note: Mean data are presented as mean±standard deviation. BMI = Body Mass Index, MOST= Multi-Center Osteoarthritis Study, OAI= Osteoarthritis Initiative. Number of patients are shown for ethnicity data.*
