# Supplementary material for: Robust disease prognosis via diagnostic knowledge preservation: A sequential learning approach
Source: PLoS One. 2026 May 6;21(5):e0344600. doi: 10.1371/journal.pone.0344600 (PMC13148697; doi:10.1371/journal.pone.0344600)
Supplement: S3 Table — (DOCX) [file pone.0344600.s004.docx]

**S3 Table**. Demographic Characteristics and Cognitive Status Distribution across the ADNI Prognosis Cohort.

| **Parameters** | **Men** | | **Women** | |
| --- | --- | --- | --- | --- |
|  | **Patients** | **Controls** | **Patients** | **Controls** |
| No. of patients | 89 | 116 | 59 | 101 |
| No. of scans | 204 | 325 | 139 | 246 |
| Age | 75.7 ± 7.3 | 75.5 ± 6.2 | 73.5 ± 7.1 | 75.2 ± 5.9 |
| Cognitive Normal (CN) | 19 | 180 | 12 | 175 |
| Mild Cognitive Impaired (MCI) | 185 | 145 | 127 | 71 |
| **Ethnicity** | | | | |
| White | 85 | 107 | 55 | 93 |
| Black | 2 | 5 | 3 | 8 |
| Asian | 2 | 4 | 1 | 0 |

Note: Data shows age (mean ± standard deviation) and number of subjects by cognitive status (CN vs MCI) for both controls (no disease progression, label = 0) and patients (Disease progression, label = 1).
