# Supplementary material for: Robust disease prognosis via diagnostic knowledge preservation: A sequential learning approach
Source: PLoS One. 2026 May 6;21(5):e0344600. doi: 10.1371/journal.pone.0344600 (PMC13148697; doi:10.1371/journal.pone.0344600)
Supplement: S4 Table — (DOCX) [file pone.0344600.s005.docx]

**S4 Table.** Breast cancer Patient distribution in the Prognosis Cohort.

| **Parameters** | **Patients** | **Controls** |
| --- | --- | --- |
| No. of patients | 2,319 | 2,837 |
| No. of exams | 3,000 | 3,000 |
| Mean age (y) | 61.5±11.4 | 56.4±10.8 |
| **Exam-level BI-RADS** | | |
| 0 | 453 | 383 |
| 1 | 962 | 1,411 |
| 2 | 1,599 | 1,189 |
| others | 26 | 17 |
| **Density** | | |
| Almost entirely fatty | 112 | 260 |
| Scattered areas of fibroglandular density | 821 | 1,200 |
| Heterogeneously dense | 1,119 | 1,124 |
| Extremely dense | 179 | 221 |
| Unknown | 88 | 32 |
| **Ethnicity** | | |
| White | 1,588 | 1,774 |
| Black | 220 | 248 |
| Asian | 115 | 159 |
| Other Nonwhite | 396 | 656 |
