# Supplementary material for: Robust disease prognosis via diagnostic knowledge preservation: A sequential learning approach
Source: PLoS One. 2026 May 6;21(5):e0344600. doi: 10.1371/journal.pone.0344600 (PMC13148697; doi:10.1371/journal.pone.0344600)
Supplement: S5 Table — (DOCX) [file pone.0344600.s006.docx]

**S5 Table.** Comparison of AUROC performance for models trained on progression and incidence, using different initializations.

| **Approach** | **Incidence (KL- 0,1)** | | | | **Progression (KL - 2,3)** | | | |
| --- | --- | --- | --- | --- | --- | --- | --- | --- |
|  | **OAI AUROC** | **OAI AUPRC** | **MOST AUROC** | **MOST AUPRC** | **OAI AUROC** | **OAI AUPRC** | **MOST AUROC** | **MOST AUPRC** |
| ImageNet pretrained | 0.697 ± 0.02 | 0.222 ± 0.017 | 0.707 ± 0.019 | 0.380 ± 0.023 | 0.726 ± 0.011 | 0.561 ± 0.016 | 0.699 ± 0.011 | 0.672 ± 0.009 |
| Diagnosis pretrained | 0.699 ± 0.009 | 0.186 ± 0.011 | 0.742 ± 0.013 | 0.414 ± 0.027 | 0.718 ± 0.011 | 0.571 ± 0.015 | 0.731 ± 0.005 | 0.722 ± 0.009 |
