# Supplementary material for: Robust disease prognosis via diagnostic knowledge preservation: A sequential learning approach
Source: PLoS One. 2026 May 6;21(5):e0344600. doi: 10.1371/journal.pone.0344600 (PMC13148697; doi:10.1371/journal.pone.0344600)
Supplement: S6 Table — (DOCX) [file pone.0344600.s007.docx]

**S6 Table.** Detailed AUROC analysis comparing model performance across cognitive status subgroups.

| **Approach** | **Within Group** | | **Cross Group** | |
| --- | --- | --- | --- | --- |
|  | **CN (0 vs 1)** | **MCI (0 vs 1)** | **CN 1 vs MCI 0** | **CN 0 vs MCI 1** |
| Random initialization | 0.681 ± 0.103 | 0.750 ± 0.028 | 0.461 ± 0.065 | 0.888 ± 0.021 |
| Diagnosis Pretrained | 0.713 ± 0.037 | 0.773 ± 0.016 | 0.507 ± 0.029 | 0.924 ± 0.009 |
