# Supplementary material for: Robust disease prognosis via diagnostic knowledge preservation: A sequential learning approach
Source: PLoS One. 2026 May 6;21(5):e0344600. doi: 10.1371/journal.pone.0344600 (PMC13148697; doi:10.1371/journal.pone.0344600)
Supplement: S7 Table — (DOCX) [file pone.0344600.s008.docx]

**S7 Table.** Detailed metrics for various training strategies for progression and incidence prediction tasks on OAI and MOST Datasets.

| **Approach** | **Incidence (KL-0,1)** | | | | **Progression (KL-2,3)** | | | |
| --- | --- | --- | --- | --- | --- | --- | --- | --- |
|  | **OAI AUROC** | **OAI AUPRC** | **MOST AUROC** | **MOST AUPRC** | **OAI AUROC** | **OAI AUPRC** | **MOST AUROC** | **MOST AUPRC** |
| Diagnosis pretrained Ref | 0.700 ± 0.017 | 0.220 ± 0.019 | 0.751 ± 0.006 | 0.433 ± 0.008 | 0.725 ± 0.003 | 0.567 ± 0.008 | 0.735 ± 0.002 | 0.731 ± 0.003 |
| Single Cohort MT | 0.686 ± 0.026 | 0.223 ± 0.033 | 0.727 ± 0.048 | 0.406 ± 0.052 | 0.721 ± 0.013 | 0.552 ± 0.012 | 0.698 ± 0.010 | 0.663 ± 0.015 |
| Concurrent MT | 0.695 ± 0.021 | 0.242 ± 0.026 | 0.736 ± 0.011 | 0.405 ± 0.017 | 0.711 ± 0.014 | 0.532 ± 0.010 | 0.714 ± 0.013 | 0.702 ± 0.009 |
| Diagnosis pretrained MT | 0.704 ± 0.009 | 0.233 ± 0.016 | 0.739 ± 0.011 | 0.422 ± 0.018 | 0.724 ± 0.006 | 0.566 ± 0.008 | 0.709 ± 0.010 | 0.715 ± 0.007 |
| Seq Learning w Replay | 0.706 ± 0.012 | 0.209 ± 0.011 | 0.735 ± 0.007 | 0.397 ± 0.018 | 0.728 ± 0.013 | 0.570 ± 0.010 | 0.718 ± 0.016 | 0.713 ± 0.016 |
