# Supplementary material for: Robust disease prognosis via diagnostic knowledge preservation: A sequential learning approach
Source: PLoS One. 2026 May 6;21(5):e0344600. doi: 10.1371/journal.pone.0344600 (PMC13148697; doi:10.1371/journal.pone.0344600)
Supplement: S8 Table — (DOCX) [file pone.0344600.s009.docx]

**S8 Table.** Detailed progression prediction performance (AUROC) across cognitive status subgroups for multitask methods.

| **Approach** | **Within Group** | | **Cross Group** | |
| --- | --- | --- | --- | --- |
|  | **CN (0 vs 1)** | **MCI (0 vs 1)** | **CN 1 vs MCI 0** | **CN 0 vs MCI 1** |
| Diagnosis pretrained Ref | 0.713 ± 0.037 | 0.773 ± 0.016 | 0.507 ± 0.029 | 0.924 ± 0.009 |
| Single cohort MT | 0.618 ± 0.053 | 0.729 ± 0.020 | 0.418 ± 0.029 | 0.897 ± 0.024 |
| Concurrent MT | 0.733 ± 0.042 | 0.751 ± 0.014 | 0.506 ± 0.028 | 0.916 ± 0.021 |
| Diagnosis pretrained MT | 0.723 ± 0.038 | 0.777 ± 0.013 | 0.501 ± 0.030 | 0.938 ± 0.009 |
| Seq learning w replay | 0.735 ± 0.040 | 0.779 ± 0.007 | 0.514 ± 0.022 | 0.939 ± 0.012 |
